# 

**Published:** 1918-10-26

**Authors:** 


### Forthcoming Events.

Tuberculosis Society: Opening of Winter Session.—Sir Malcolm Morris, K.C., V.O., will give an address on "Past and Future of the Fight against Tuberculosis" on Monday, October 28, at 8 p.m., within the Royal Society of Medicine, 1 Wimpole Street, London, W. The meeting is open to all interested.

### Matrons' and Sisters' Appointments.

Grata Quies Auxiliary Hospital, Bournemouth.—Miss R. Winifred Brayley has been appointed sister. Particulars of her training were given in our issue of the 19th inst.

Infectious Diseases Hospital and Tuberculosis Sanatorium, Stone.—Miss Eva Wilcox has been appointed matron. She was trained at Fulham Infirmary, has been matron of Runcorn Hospital, and later joined the Scottish Women's Hospitals for Foreign Service.

### Village Life and Industries.

The Exhibition of Women's Work at Caxton Hall on October 25 to 29 inclusive is to be honoured by a visit from the Queen. It will be of special interest as showing what women in the country have done since their interest has been awakened and proper training given in cookery, vegetable and fruit-growing, canning, and preserving, the care of rabbits, curing of skins, basket-making and other crafts, laundry and home dyeing, dress-making, toy-making, use of waste products, etc., etc.

All persons in uniform will pay 8d. admission only.

### The Duchess's Retort.

A woman, one of 30,000 British working for the Y.M.C.A., was assigned to scrubbing the Eagle Hut floor. She had done little manual work in her life, but accepted the job without protest, and went down on her knees with a pail of hot water, a cloth, and a cake of soap. Soon the water in the pail was black. A man in uniform passed. The woman looked up and asked if he would mind emptying the pail and refilling it with clean water.

There was a theatrical pause, then this reply:

"Damn it, madam, I'm an officer!"

This time there was no pause, but like a flash the scrubwoman retorted:

"Damn it, officer, I'm a duchess!"—*Sunday Times.*

